# Ten Years of the Collaborative Cross

**DOI:** 10.1534/g3.111.001891

**Published:** 2012-02-01

**Authors:** David W. Threadgill, Gary A. Churchill

**Affiliations:** * Department of Genetics, North Carolina State University, Raleigh, North Carolina 27695; † The Jackson Laboratory, Bar Harbor, Maine 04609

## Abstract

The February 2012 issues of GENETICS and G3: Genes, Genomes, Genetics present a collection of articles reporting recent advances from the international Collaborative Cross (CC) project. The goal of the CC project is to develop a new resource that will enhance quantitative trait locus (QTL) and systems genetic analyses in mice. The CC consists of hundreds of independently bred, octo-parental recombinant inbred lines (Figure 1). The work reported in these issues represents progress toward completion of the CC, proof-of-principle experiments using incipient inbred CC mice, and new research areas and complementary resources facilitated by the CC project.

**Figure 1:**
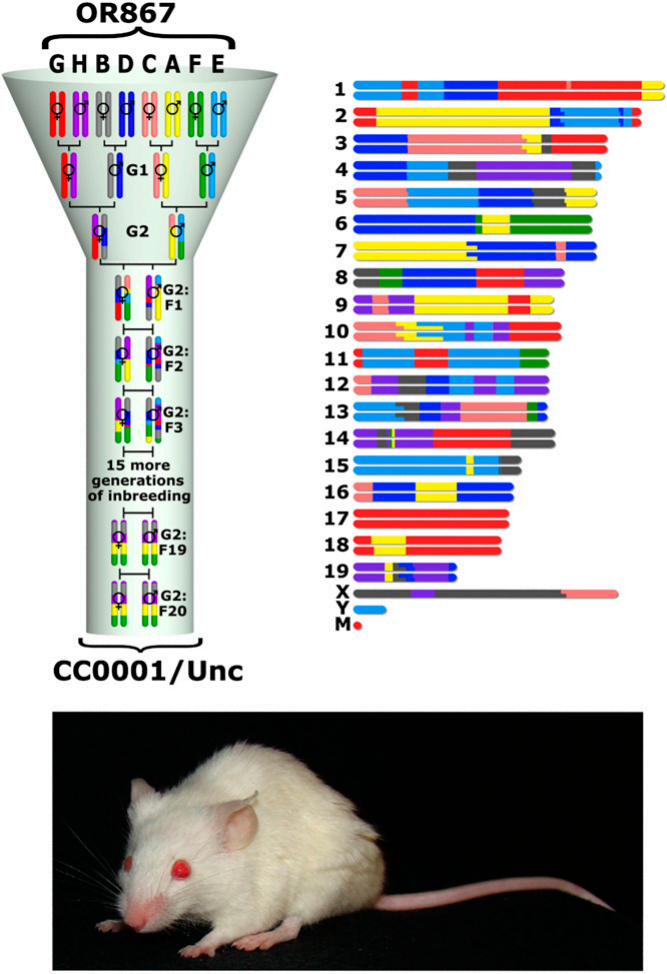
Development of an individual CC line. Each CC line originates from an independently breeding funnel so that every recombination site in the CC population is uniquely generated. In this example, a CC line is produced from the breeding line with laboratory code OR867. The first CC line to be declared inbred was from this breeding funnel and was given the official designation CC0001/Unc. A representation of the genome color-coded by founder strain contribution and a photograph of a mouse from CC0001/Unc is shown next to the breeding funnel.

## Collaborative Cross Past

The idea to develop a multiparental recombinant inbred panel was conceived more than 10 years ago in response to a challenge positing that discovering the genetic basis of complex traits would be more efficient using genome-wide mutagenesis (Nadeau and Frankel 2000), which at the time was undergoing a renaissance. Subsequently, the concept for a new, purpose-built mouse resource was presented at a satellite meeting of the 15th International Mouse Genome Conference in Edinburgh, Scotland (Threadgill *et al.* 2002). The Complex Trait Consortium (CTC; since renamed Complex Trait Community) was also established during the satellite meeting.

A committee from the CTC met the following year to debate the structure and specifics of a breeding design that would mix and randomize multiple genomes into an experimental population where each recombination site was unique and whose size was sufficiently powered to support QTL analysis at near single-gene resolution (supporting information, File S1). Because of community involvement in designing and implementing this new resource, it became known as the Collaborative Cross (Churchill *et al.* 2004). Subsequent discussions with the broader genetics community lead to the realization that the CC would be far more than a new QTL mapping resource; a resource that could support integrative, systems-level biology or “systems genetics” in mice (Threadgill 2006).

After extensive discussions to determine which extant inbred strains (the “founders”) would be used as the initiating germplasm, eight strains were selected from existing laboratory and wild-derived strains representing the three major *Mus musculus* subspecies (*M. m. musculus*, *M. m. domesticus*, and *M. m. castaneous*): A/J, C57BL/6J, 129Sv/ImJ, NOD/LtJ, NZO/H1J, CAST/EiJ, PWK/PhJ, and WSB/EiJ. Genetic analysis revealed that the eight strains captured almost 90% of the known genetic variation present in laboratory mice originating from *Mus musculus* and that the captured variation is randomly distributed across the genome (Roberts *et al.* 2007).

The CC project was formally initiated in 2004 at The Jackson Laboratory through the generation of a full, reciprocal diallel cross among the eight founder strains. The resulting F1 population was shipped to three breeding sites, Oak Ridge, Tennessee (Oak Ridge National Laboratory); Perth, Australia (Western Australian Institute for Medical Research/Geniad Ltd.), and Nairobi, Kenya (International Livestock Research Institute), for two subsequent crosses to generate octo-parental mice from which inbred CC lines were developed. The U.S. population was eventually moved to Chapel Hill, North Carolina (University of North Carolina) and the Kenyan population to Tel Aviv, Israel (Tel Aviv University). Interim reports from the three major breeding centers were previously published (Chesler *et al.* 2008; Iraqi *et al.* 2008; Morahan *et al.* 2008).

During the 10 year journey to develop the CC, many additional research areas were facilitated, including analysis and simulation of expected numbers of recombination sites and the rate of inbreeding in multi-parental inbred lines (Broman 2005), and QTL mapping simulations to predict the eventual power of the CC (Valdar *et al.* 2006). Unanticipated challenges, such as the observation that the majority of initiated CC lines become extinct, were also encountered.

## Collaborative Cross Present

Initial genetic and phenotypic analyses of the CC revealed the absence of large-scale selection within the CC population; balanced founder allele frequencies; dense, evenly distributed recombination sites; and phenotype distributions exceeding other extant resources (Aylor *et al.* 2011; Philip *et al.* 2011). Mapping resolution of expression QTL (eQTL) was shown to be ≤1 Mb using as few as 156 mice from incipient inbred CC lines (called pre-CC mice). Analysis of the CC genome architecture has been greatly extended in an article from the Collaborative Cross Consortium in GENETICS (2012). The authors report a detailed genetic analysis of extant CC lines from the three breeding populations. This analysis revealed a small number of lines with breeding errors, such as missing founders, but also confirmed previous analysis showing that the founder haplotypes are inherited at expected frequencies. Evidence of transmission ratio distortion shared in all three independently breeding CC populations is also presented, suggesting a biological basis rather than a husbandry cause. The three breeding sites used different husbandry practices ranging from computer-assisted randomization of brother−sister matings to harem sibling matings and within-lineage backcrossing. The most important finding from this work was the absence of gametic disequilibrium in the CC population. Inter-chromosomal associations are extensive in other inbred strains, which is the underlying cause for high false-positive rates in genome-wide association studies when using the existing populations of mouse strains (Payseur and Place 2007).

Monitoring the inbreeding rates within CC lines confirmed that an extended number of generations would be needed to reach inbred status for many of the CC lines (Broman 2005). An article in this issue of G3 by Welsh and McMillan reports the development of genotype-driven, accelerated inbreeding technique that is being exploited to overcome the lengthy process needed to achieve fixation of regions with heterozygosity (2012). Simulations demonstrated that the number of generations to reach inbred status using randomized sib-mating can be substantially reduced with minimal impact on the number of retained recombination sites by using a combination of parental backcrossing and marker-assisting inbreeding. The number of generations can be reduced by almost ten, while reducing the number of recombination sites in the final line by < 5%. The marker-assisted inbreeding approach is currently being used to complete the inbreeding of CC lines from all three breeding sites. Until all CC lines are inbred, many investigators are using pre-CC mice from intermediate generations in proof-of-principle experiments. For example, previous studies have mapped and proposed candidate genes for susceptibility to *Aspergillus fumigatus* infection and energy balance using pre-CC mice (Durrant *et al.* 2011; Mathes *et al.* 2011).

The power and resolution of mapping studies using pre-CC mice is further demonstrated in articles in G3 by Kelada *et al.* and Bottomly *et al.* (2012). Kelada *et al.* (2012) elucidated genetic factors controlling hematological parameters including red blood cell volume, white blood cell count, percentage of neutrophils, and monocyte number. They proposed a number of candidate genes by narrowing QTL intervals in the context of phylogeny. Similarly, Bottomly *et al.* (2012) used pre-CC mice to elucidate the genetic control of eQTL in mice with extreme response to Influenza A infection. These studies employ regression analysis on eight founder haplotypes (Aylor *et al.* 2011). The pattern of allele effects across the eight haplotypes used in conjunction with whole- genome sequences provides a greatly reduced set of candidate SNPs in contrast to two-way crosses where all segregating SNPs are candidate causal loci.

Analysis methods for pre-CC mice remain an area of rapid development. In an article by Broman in GENETICS, a new approach using *k*-step probabilities for a Markov chain on genotype states is presented to infer genotype probabilities at intermediate stages of inbreeding (2012a). In addition to the elegant mathematics used in their derivation, these probabilities have utility for analyses of pre-CC populations. Computational methods presented in a an article by Z. Zhang *et al.* in this issue of G3 also improve the analysis of pre-CC data (Z. Zhang *et al.* 2012). Zhang *et al.* (2012) presents a new association mapping technique using semi-perfect phylogenetic trees that make allowance for heterozygous regions found in pre-CC mice. They validate the analysis using the Mendelian trait “white spotted blaze” that segregates in the CC population from the WSB/EiJ founder and the quantitative phenotype running distance.

The Diversity Outbred (DO), a complementary resource to the CC, was initiated at The Jackson Laboratory using CC lines from the U.S. population at intermediate stages of inbreeding. An article in GENETICS by Svenson *et al.* provides the first description and genetic analysis using the DO population (2012). The DO is a population of mice with the same genetic composition as the CC but maintained outbred through random matings, which increases mapping resolution through accumulation of additional recombination sites. Methods for analyzing the multi-allelic outbred DO population are presented and validated using the identification of *Foxo1* as a candidate for a QTL regulating serum cholesterol. A second analytical article by Broman in G3 (Broman 2012b) provides key results for computing genotype probabilities in DO mice. An analysis of a commercial outbred stock, presented in a G3 article by W. Zhang *et al.* underscores the lack of genetic diversity in current mouse stocks that motivated the creation of the CC and DO populations (W. Zhang *et al.* 2012). Data presented in these papers confirm and extend the power of a previous heterogeneous stock also developed from the CC founders (Iancu *et al.* 2010).

Numerous other advances in complex trait analysis have been facilitated by the CC project. An article by Wang *et al.* in GENETICS describes a new method to impute SNPs using local phylogeny trees (Wang *et al.* 2012), which uses whole-genome sequences from 17 inbred strains including the eight CC founders (Keane *et al.* 2011). Imputed genomes were generated for 100 extant mouse strains resulting in almost one billion new genotype calls with less than 0.4% error genome-wide. This work greatly extends previous work to use genome imputation for genome ancestry in complex inbreeding pedigrees (Liu *et al.* 2010), and will support genetic and genomic analyses of extant inbred strains whose genomes have not been sequenced.

Another advance reported in GENETICS is an improvement in the analysis of diallel crosses, which have been previously used to characterize the aggregate effects of genetic background on phenotypes. The article by Lenarcic *et al.* analyzes phenotypic data obtained on mice from the diallel of founders used to initiate production of the CC. They employ a new Bayesian method for analysis of diallel crosses that improves decomposition of phenotypic variance into biologically intuitive components (Lenarcic *et al.* 2012).

## Collaborative Cross Future

The CC will become the primary platform for systems-level analysis in mammals. Using the brain as a model multi-compartment organ, Sun *et al.* report in an article in G3 that inter-strain variation in gene expression is greater in discrete brain regions compared to inter-strain transcriptional differences detected comparing whole brains (Sun *et al.* 2012). This study has important implications for future whole-body dissections of CC mice for integrated systems analysis. The CC will continue to be an important stimulus for supporting QTL and systems genetics research. The CC has already proven to be a superior population for selective breeding (Zombeck *et al.* 2011), and will likely be exploited for future genetic selection experiments targeting specific phenotypes.

Future applications of the CC will exploit the strategy of recombinant inbred intercrosses (RIX), which are diallel crosses using panels of recombinant inbred lines (Zou *et al.* 2005), to substantially extend the numbers of unique genotypes. Compared with inbred lines, RIX have statistically lower phenotypic variances. In addition, RIX populations support analysis of parent-of-origin and transgenerational effects which will further our understanding of genetic causes of phenotypes with complex etiologies. An article by Gong and Zou in GENETICS builds upon previous RIX studies by presenting a flexible, nonparametric time varying coefficient QTL mapping method for RIX data (Gong and Zou 2012). This work will support future use of the CC to investigate gene-by-time interactions as well as the genetic control of phenotypic variance as opposed to the classical analysis of phenotypic means (Ronnegard and Valdar 2011). Understanding genetic control of phenotypic variance is essential for improving the accuracy of predictive medicine.

Generation of RIX will further support investigations into the genetic architecture of extinct CC lines aimed at elucidating the causes of genome incompatibilities, which has important implications for reproductive medicine. The extinction that occurred in many individual CC lines could be attributable to fixation of incompatible alleles at different loci, presumably originating from the three *M. musculus* subspecies. Identifying homozygous allele combinations in extinct lines that are not observed in the extant CC lines will provide candidates that can be synthetically tested by recreating unique allele combinations using specific RIX from CC lines.

More than a decade after the conception of the Collaborative Cross, the first fully inbred strains are becoming available for researchers to use. The lead paper in GENETICS 190(2) presents a model for community access to the CC resource (Collaborative Cross Consortium 2012). Maintenance and distribution of the new lines will present challenges that require ongoing community support. If the CC is to deliver on its promise, it must be used. In-depth characterization of phenotypes spanning multiple domains of mammalian biology will be needed to develop a systems-level perspective. New databases and analytical tools are already under development (http://compgen.unc.edu/wp/ and http://churchill.jax.org/research/cgd.shtml). Experimental designs that combine the CC, DO, and other resources, such as the comprehensive mouse knock-out panel, are being proposed to extend the scope of investigations using the CC strains. The energy and ingenuity of the community of users who have been awaiting the arrival of this resource is ready to be unleashed. The articles in the current issues of GENETICS and G3 provide just a glimpse of what is to come.

## Supplementary Material

Supporting Information
