# Supplementary material for: Ten Years of the Collaborative Cross
Source: G3 (Bethesda). 2012 Feb 1;2(2):153–6. doi: 10.1534/g3.111.001891 (PMC3284322; doi:10.1534/g3.111.001891)
Supplement: Supporting Information [file supp_2_2_153__index.html]

Supporting Information 

# Ten Years of the Collaborative Cross

## Supporting Information for Threadgill and Churchill, 2012

**Files in this Data Supplement:**

- File S1 - Complex Trait Consortium - 1st Workshop Report: September 2002 (PDF, 1 MB)
