# Supplementary material for: Ten Years of the Collaborative Cross
Source: G3 (Bethesda). 2012 Feb 1;2(2):153–6. doi: 10.1534/g3.111.001891 (PMC3284322; doi:10.1534/g3.111.001891)
Supplement: Supporting Information [file supp_2.2.153_FileS1.pdf]

Complex Trait Consortium

1st Workshop Report: Sept 2002

A Collaborative Cross for High-Precision Complex Trait Analysis

RW Williams, KW Broman, JM Cheverud, GA Churchill, RW Hitzemann, KW Hunter, J Mountz, D Pomp, RH Reeves, LC Schalkwyk, DW Threadgill

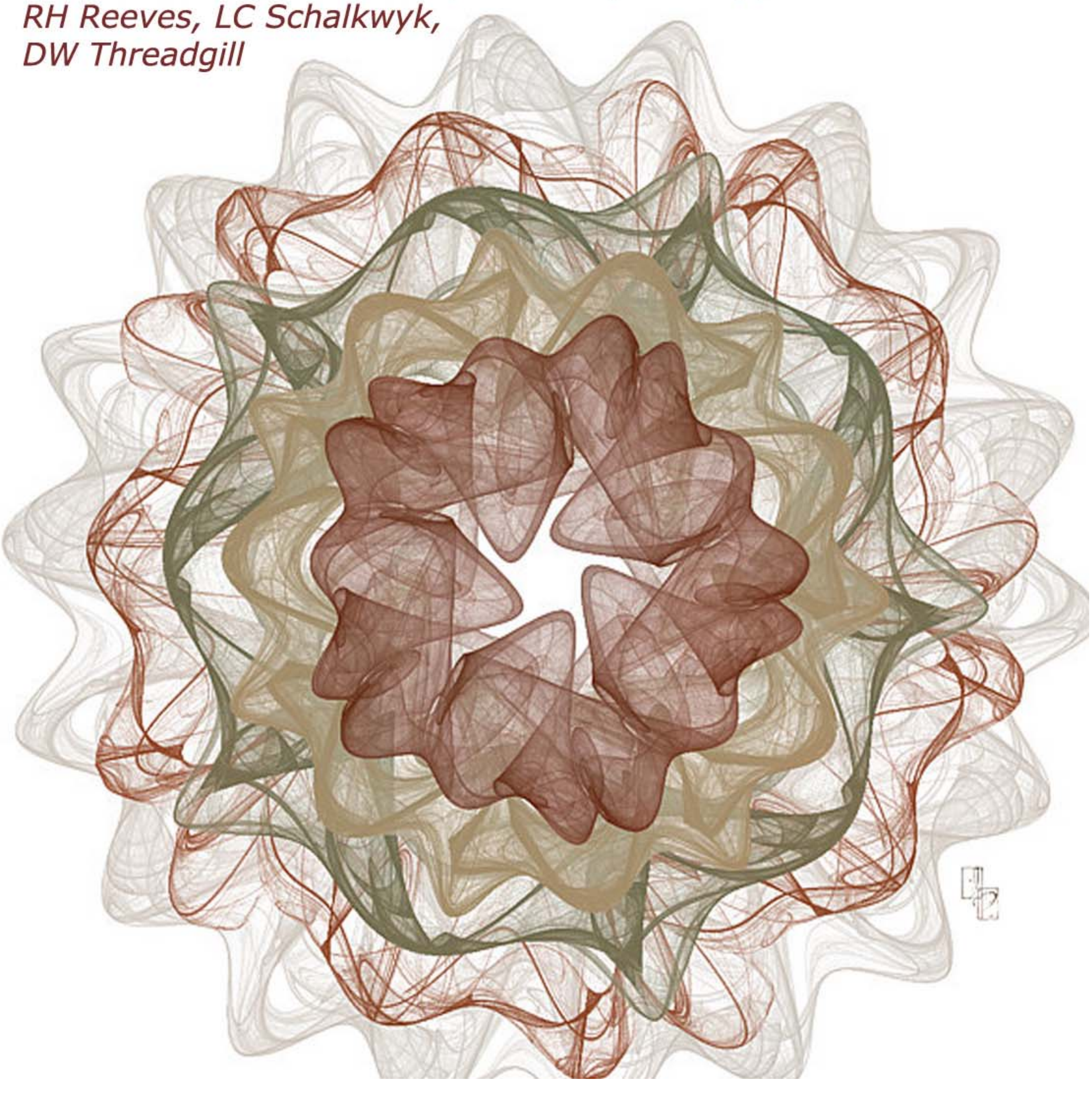

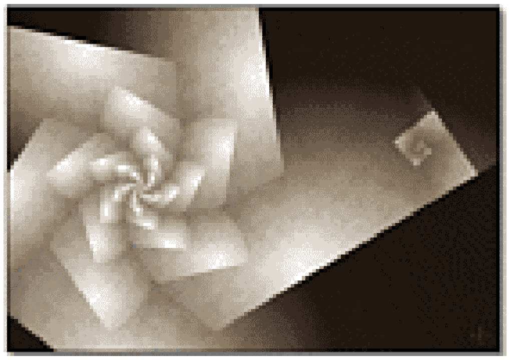

Cover: by Janet Parke. Modified from Vikon fractal.
www.infinite-art.com
Reproduced with permission.

At Genetic Frontier, the House Mouse Serves Humanity
NY Times, Dec 10, 2002, by Nicholas Wade

"There is also a Complex Traits Consortium, whose aim is to find the genetic roots of the many important diseases that are caused by several genes acting in concert. That goal has long eluded human geneticists, and the consortium hopes to achieve the feat in the mouse."

Please cite as

Williams RW, Broman KW, Cheverud JM, Churchill GA, Hitzemann RW, Hunter KW, Mountz JD, Pomp P, Reeves RH, Schalkwyk LC, Threadgill DW (2002) A collaborative cross for high-precision complex trait analysis. 1st Workshop Report of the Complex Trait Consortium: Sept 2002. www.complextrait.org/Workshop1.pdf

---

This workshop was organized and hosted by Karl Broman and Roger Reeves. Participants are all members of the CTC, an international group that consists of ~150 geneticists.

v 31 (July 2003)

A Collaborative Cross for High-Precision Complex Trait Analysis

CTC Workgroup Report

Robert W. Williams, Karl W. Broman,
James M. Cheverud, Gary A. Churchill,
Robert W. Hitzemann, Kent W. Hunter,
John Mountz, Daniel Pomp, Roger H. Reeves,
Leonard C. Schalkwyk, David W. Threadgill

"Most genetic traits of interest in populations of humans and other organisms are determined by many factors, including genetic and environmental components, which interact in often unpredictable ways. For such complex traits, the whole is not only greater than the sum of its parts, it may be different from the sum of its parts. Thus, complex traits have a genetic architecture that consists of all of the genetic and environmental factors that contribute to the trait, as well as their magnitude and their interactions."

National Institute of General Medical Sciences, 1998,
Complex Trait Workshop Report
www.nigms.nih.gov/news/reports/genetic_arch.html

Abstract

The goal of the Complex Trait Consortium (CTC) is to produce greatly improved resources that can be used to understand, treat, and ultimately prevent the most pervasive human diseases. Essentially all human diseases are complex in the sense that incidence, severity, and outcome are determined by interactions among many genes and environmental factors. Cancer, diabetes, heart and lung disease, Alzheimer's, and infectious diseases all fall into this category. Members of the CTC have spent the past year devising a detailed plan to generate a single collaborative genetic resource that will greatly accelerate the study of human disease. The resource is called the Collaborative Cross. This Report explains our reasoning and provides an outline of the methods and costs associated with making the Collaborative Cross.

Introduction to the Collaborative Cross

Eleven members of the Complex Trait Consortium (CTC) met at Johns Hopkins University, August 30, 2002, to refine ideas that were initially proposed at CTC meetings in Edinburgh (Nov 2001) and Memphis (May 2002). We focused on how to move forward on a large-scale collaborative project to provide biomedical researchers with much more powerful resources for systematic analysis of complex traits. We plan to submit proposals to support the Collaborative Cross in the USA, Europe (Leo Schalkwyk and colleagues), and Australia (Grant Morahan and colleagues) in 2003. At least seven years of funding will be required to meet initial goals.

A summary of work that led to the meeting at Johns Hopkins is provided in a recent paper by Threadgill and colleagues (2002). Additional papers, abstracts, and pdf files (including this report) are available online at *www.complextrait.org.* Gibson and Mackay (2002) have summarized work to produce more powerful community resources for evolutionary genomics that in many ways parallel and complement our CTC efforts.

The Baltimore workgroup explored alternative methods to produce a large multipurpose set of recombinant inbred (RI) strains of mice. We settled on a cross that will combine eight inbred strains and that will therefore be useful for the analysis of virtually any murine complex trait. The plan summarized in this report is still provisional, and we solicit your input and criticism. The selection of strains is particularly critical, and this open topic is considered in more detail below. We have opted to select strains that provide the greatest genetic diversity. In all likelihood, some preference will be given to several strains that are widely used by the community.

While the workshop dealt specifically with our proposal to generate a large set of RI strains, many CTC members are actively developing complementary methods to study complex traits. The Collaborative Cross is not intended to supplant other work or the development of other resources. However, the consensus of the CTC is that this particular project should have highest priority.

This report is divided into three parts. The first part summarizes aims of the resource aims and answers the question: What will the RI cross accomplish in terms of precision and power, and how will it be used by the biomedical research community? The second part summarizes a specific implementation of the Collaborative Cross. This section incorporates initial results of simulations and calculations concerning our recommended implementation. The third part is an outline of how a large CTC effort leading to the Collaborative Cross can be structured and distributed across a diverse and international community of researchers using mice in a wide variety of research projects.

PART I. AIMS AND REQUIREMENTS

In this section we briefly review nine aims that were considered in designing a single integrated collaborative cross for complex trait analysis.

1. **Broad utility**
2. **High precision, high power mapping**
3. **Appropriate models for complex human diseases**
4. **Mapping modifiers of knockouts and mutations**
5. **Powerful analysis of epistatic interactions**
6. **Utility to study gene-by-environment interactions**
7. **Effective analysis of genetic correlations**
8. **Freedom from genotyping**
9. **Unlimited access to strains, tissues, and data**

> *Note:* Some of the material that follows is technical and assumes familiarity with new mapping techniques, in particular, the RIX design. Please refer to Appendix *figure 5* for a graphic summary of several experimental crosses and mapping resources.

1. Broad utility. We expect the Collaborative Cross to be useful to essentially all researchers using rodent models and to the great majority of researchers and clinicians studying complex human diseases. For this reason, the Collaborative Cross is designed to incorporate a broad spectrum of natural allelic variants that have been sequestered within inbred strains over the past several centuries. We have settled on a cross that combines eight strains (an 8-way cross) as a

reasonable compromise between embracing allelic diversity while coping with computational and statistical challenges of a multiallele cross. The expectation is that an optimal set of eight strains will sample >80% of the allelic diversity among *Mus musculus* strains and subspecies. In addition to common allelic variation, the optimal set should also capture and have the power to dissect rare gene variants.

A recent analysis of haplotypes of several major strains and subspecies of mice (Wade et al., 2002) emphasizes that some of the most common inbred strains are hybrids of two or three subspecies of mice (www.nature.com/nature/mousegenome/). Their genomes typically consist of alternative haplotype blocks inherited from two or three ancestral mouse subspecies. In contrast, the often marked phenotypic differences between closely related substrains of mice, for instance, BALB/cJ and BALB/cByJ, highlights the rapidity with which additional gene variants and chromosomal alternations can be accumulated in laboratory mice. By combining eight strains into a single cross we gain a tremendous resource in the form of both the haplotype breakpoints that have been archived in each inbred strain, as well as the novel alleles fixed within the past few hundred generations of inbreeding in each of eight lines.

2. High precision, high power mapping. The Collaborative Cross is a resource that is designed to achieve 0.1 cM precision—equivalent to approximately 200,000 basepairs of DNA—when mapping the types of QTLs with which most investigators now routinely work (additive effects of > 0.25 SD). This level of precision will exist in a single well geno-typed cross. It will not be necessary to generate or genotype custom secondary mapping resources. Achieving this level of precision with high statistical power in a single mapping panel requires archiving approximately 100,000 independent recombination breakpoints in the set of strains. The critical issue of statistical power also makes it necessary to generate large numbers of strains. A set of 1000 strains containing 100,000 breakpoints is a far more powerful research tool than 100 strains containing the same number of breakpoints.

In addition to recombination breakpoints that we will introduce systematically by intercrossing, there are also large numbers of historical haplotype breakpoints in each of the parental strains. This second, deeper level of ancestral breakpoints will be of utility in fine-grained analysis of QTL position (for an example of this new approach see Hitzemann et al., 2002). We expect the combination of new and ancestral breakpoints will often give QTL positional resolution of <100 kbp; an interval that will contain 1 to 5 positional candidate genes.

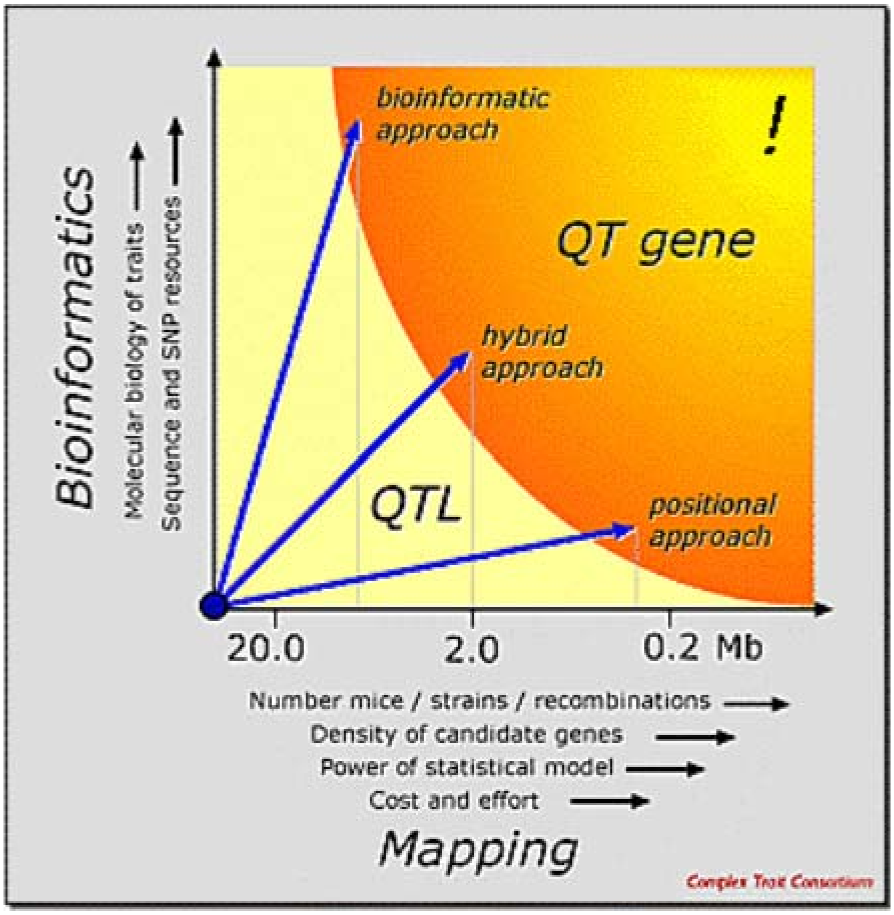

Figure 1. The conversion of QTLs into QTGs typically requires excellent positional precision and strong bioinformatic resources. The Collaborative Cross is designed to routinely and efficiently achieve high precision (0.2 to 2.0 Mb). It will also have strong and unique bioinformatic resources, including extensive data on transcriptional activity, protein abundance, and thousands of phenotypes for hundreds of lines.

Most QTL studies begin with an initial step of QTL discovery and low precision QTL mapping that is followed by one or two additional stages of mapping designed to achieve higher and higher precision (e.g., Darvasi, 1998). As a practical matter, a QTL mapping study using the Collaborative Cross will also need to be conducted using a two or three stage *step-down* approach. In the first stage, a set of 20 to 100 RI strains are phenotyped to locate the approximate positions a small number of major QTLs. The 2-LOD support intervals of these QTLs will often be in the range of 2 to 20 cM. The more refined analysis of each of these QTLs involves a second stage of analysis that exploits a different set of 10 to 20 RI strains (but from the same Collaborative Cross) all of which have recombinations in targeted chromosomal intervals. This not only tests each of the QTL positions, but simultaneously refines QTL position. The second stage should usually achieve a resolution of 0.25 to 2 cM, or may in some cases resolve two QTLs in an interval initially thought to contain only a single

QTL. In the third stage, all strains with relevant recombinations and haplotypes in strong candidate QTL intervals are phenotyped to achieve sub-millimorgan precision. Provided that sufficient numbers of strains and pre-mapped recombinations are available in the Collaborative Cross, millimorgan precision should often be reached in less than one year.

How many mice will I need to phenotype? The number of individuals that need to be phenotyped per line is a function of the heritability, the number of QTLs, and of course, the number of strains available from stock and at a reasonable cost (Belknap 1998). A Mendelian trait is most efficiently mapped using only a single animal from each of a large number of strains. In contrast, a trait involving an incidence, LD50, or variability score might require a sample size of 10 to 40 per line. The number of animals that are typed will often be influenced by factors other than efficiency of gene mapping. In general, it is better to study fewer individuals and larger numbers of strains than more individuals per line and smaller numbers of strains.

Most research groups using the Collaborative Cross are likely to start by phenotyping a subset of 100 to 200 RI, or better yet, RIX hybrids, chosen because of their ready availability, preexisting phenotype data, and known haplotypes. This subset will often consist of the isogenic RIX progeny that can be generated quickly by the major CTC breeding centers from a smaller subset of RI strains. Based on results of this first tier of analysis, investigators will then usually selectively phenotype additional animals. The large size of the potential pool of strains makes it possible to map a Mendelian trait to 1 millimorgan (~200 kb) using fewer than 200 animals by applying the two-stage step-down procedure in which the first 100 mice provide 1–2 cM precision whereas the second set of 100 mice with known recombinations within that 1–2 cM critical interval provide < 1 mM precision. This same protocol is applied to QTLs, but a three-stage method will often be more effective to reduce numbers of animals and strains that need to be phenotyped.

3. Appropriate models for complex human diseases.

The focus of human genetics is shifting from Mendelian traits to complex diseases (*Fig. 1*). Understanding why humans differ so greatly in their genetic vulnerability is a major challenge, and finding answers quickly and at a reasonable cost is vital if functional genomics is to live up to its promise. The key contribution that the Collaborative Cross will make is sets of precisely mapped QTLs that can be transferred directly to the human gene map. The resource will be a shortcut to many of the most relevant human candidate genes. This will improve the efficiency and yield of human linkage analysis and association studies.

There are many situations in which we need to know more about how environmental factors—diet, stress, infection, and environmental toxins—interact with gene variants. Factors can

be added and subtracted singly or in combination in mice to test causal relations and to expose additional gene variants that might otherwise remain hidden in the background.

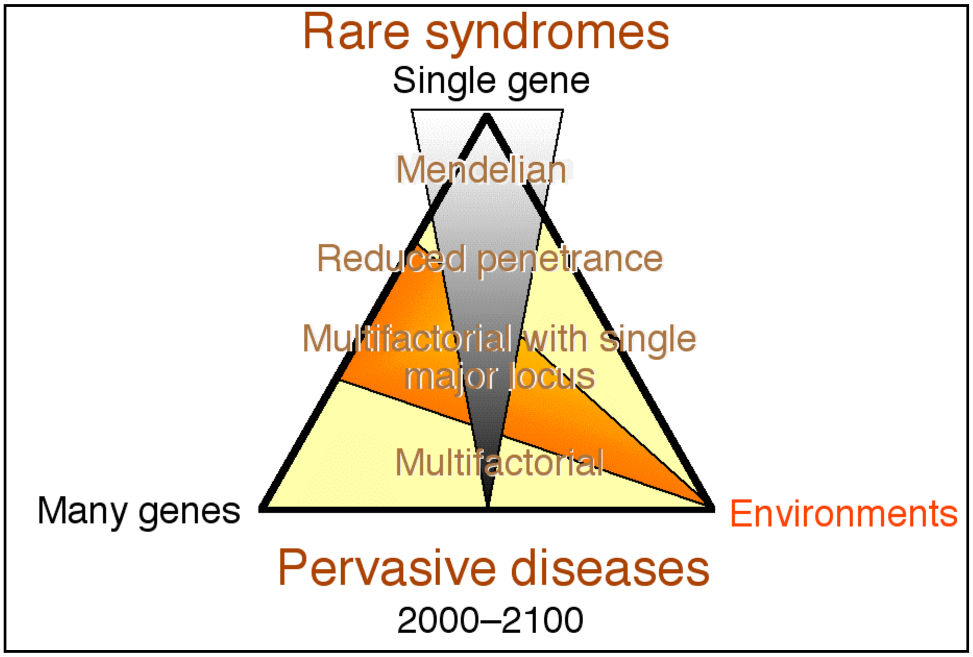

Figure 1. A simple triangular model of relations among Mendelian single gene traits (upper vertex), polygenic traits (left vertex), and multiple environmental factors (right vertex). Virtually all complex traits and most pervasive human disease occupy the base of the triangle (labeled *Multifactorial*). This base represents interactions between many genes and many environments. Modified from Strachan and Reed (1996).

Isogenic but non-inbred lines. We want to retain the advantages of mouse experimental genetics, including large sample size, known genotypes, and replicated genomes. But we would also like to be able to model the complex and non-inbred genetic structure of human populations more faithfully. The Collaborative Cross makes this possible for the first time. One of the most persuasive advantages of RI strains is the ability to map traits using isogenic lines and to study genetic correlations among thousands of traits under various environmental conditions. Although RI strains are fully inbred, this limitation can be easily sidestepped by systematically producing isogenic F1 intercrosses from the RI parents. These F1 hybrids are called RIX lines. RIX hybrids made using 8-way RI stock are almost fully outbred. Thus, an RIX mapping panel has some similarity to typical human populations in consisting of a complex and non-inbred admixture of ancestral genomes. Relevance to complex human diseases is likely to be improved relative to largely inbred mouse mapping populations.

What is an RIX line? Recombinant inbred intercross or RIX progeny are the first generation or F1 offspring made by mating

two different RI strains. For example, when RI strain AXB1 is crossed to stain AXB2, the F1 progeny are referred to as the 1x2 AB RIX. Despite the fact that 1x2 and 2x1 RIX progeny are isogenic (genetically identical) F1 animals, a group of different RIXs (1x2, 3x4, 5x6, etc) has a genetic structure that is remarkably similar to that of an F2 intercross with the classic 1:2:1 ratio of *AA*, *AB*, and *BB* genotypes. It is useful to regard single RIX lines as clones of F2 individuals. All of the advantages of RI strains apply with equal force to RIX progeny. However, RIX progeny have numerous additional advantages described briefly in Threadgill et al.(2002) and Hunter and Williams (2002; *figure 5*). These advantages include:

1. Twice the number of recombinations in a single RIX compared to a single RI strain. This fact can be exploited to gain QTL positional precison.
2. The ability to evaluate parental effects by making reciprocal RIX strains. Unlike conventional reciprocal F1s, RIX lines 1x2 and 2x1 have the same sex chromosomes and mitochondrial genomes and differ only in polarity of parental strains. Parental effects often add significant noise when measuring phenotypes and this can usually only be controlled by embryo transfer experiments. But the RIX design allows parental effect to be assess simply by generated the reciprocal crosses.
3. Dominance effects can be estimated and exploited.
4. A massive expansion of numbers of RIX compared to RI parental strains (256 RI strains can be converted into 256 x 255 RIX or 65,280t RIX lines all of which have unique and entirely defined genomes). The potential gain of statistical power is enormous.

There are also several advantages of RIX lines compared to the more usual F2 design:

1. Much higher recombination density; a single RIX line will have four times as many recombinations as a single F2 animal.
2. Genotypes are known in advance.
3. RIX strains are far more useful for long term collaborative research because they are a renewable resource.
4. Phenotype data are cumulative, enabling much more powerful multitrait complex trait analysis.

4. Mapping modifiers of knockout, transgenic, and induced mutations. The resource must be useful to map the multitude of gene variants that affect the penetrance and action of major effect alleles produced experimentally. Modifiers of both dominant and recessive alleles can be very efficiently mapped by crossing knockout, transgenic, and mutant stock to a series of the RI strains. In the great majority of cases, there will be significant interactions with alleles at several loci that segregate in the Collaborative Cross. This

produces sets of isogenic backcross strains that are ideal for high precision phenotyping by many investigators. A set of 20 to 30 RI backcross (RIB; see Hunter and Williams 2002), can be used to map modifiers, again without any genotyping. When the strong allele is recessive, the RIB progeny are intercrossed to recover homozygotes. A set of RIB X RIB progeny from 10–20 parental RIs can be treated as individual groups, making it possible to map modifiers again without any genotyping. Optionally, the RIB x RIB progeny can be genotyped for increased power and precision.

Genetic background effects are pervasive and potentially highly informative in understanding Mendelian loci. Having a single well phenotyped and well genotyped panel of RI strains incorporating many common modifier alleles will greatly enrich functional genomics of knockout, transgenic, and mutant alleles. It will be possible to systematically extract modifiers without any genotyping and with simple one-generation crosses. This approach will be unusually powerful when the Collaborative Cross when differences in transcripts and proteins have been characterized in several organs and tissues.

5. Analysis of epistatic interactions. The need to detect and characterize epistatic interactions systematically is a major factor that has driven our recommendation for a large number of strains in the Collaborative Cross. With an 8-way cross, a private allele (an allele present in only one of the eight strains) will typically appear in 32 of 256 RI strains. Four RI strains will typically be homozygotes at any two unlinked loci in the desired combination. This small sample size is clearly not sufficient to test epistatic interactions among private alleles. In contrast, a set of 1024 RI strains (a 1K set) leads to much higher *Ns* and excellent prospects for analysis of epistasis. The 1K set will typically include at least 16 two-locus RI strains and 120 derivative RIX lines. For biallelic loci (four *A*-type and 4 *B*-type alleles in an 8-way cross) three- and even four-way interactions can also be explored effectively. For example, in a best-case scenario, 4-way epistatic interactions can be tested in 16 RI strains and 120 RIX derivatives. The obvious point to make here is that epistasis, as well as mapping precision, are major factors motivating the need for high numbers of strains. Cloning QTLs will provide an important advantage when dissecting epistasis. Analysis is simplified once one partner of an interaction model has been defined precisely.

QTL designer mice. With a large set of well genotyped RI strains one can synthesize cohorts of isogenic but non-inbred RIX strains that have specific multi-allele genotypes—what one might call *QTL designer mice*. For example, if three QTLs

appear to have additive effects on a trait, then one will often be able to intercross the correct sets of RI strains to generate RIX progeny with specific combinations of alleles at all three loci in a single generation.

What is the cost of such a large strain count? Maintaining large number of strains must be done efficiently and within a reasonable budget. We therefore think that a two-tiered colony system should be employed to allow the community to retain large numbers of strains while minimizing cage costs. One solution would be to maintain a set of 256 RI strains (a consensus set) in a “readily available” category (equivalent to Jackson Laboratory category 3). Virtually any RIX from these RIs would be available within two months. The second tier would consist of all other viable RI strains. These strains would be retained at the lowest number of cages consistent with idiosyncratic breeding performance (category 4). Strains with marginal breeding performance would be relegated as soon as possible to cryopreservation (category 5) or would simply be discarded after harvesting tissues, cell lines, and when possible ES cell lines.

We estimate that it will be practical to maintain all viable strains (preferably a full set of 1024 strains) for under $1.0 M a year across the entire consortium. Our estimate assumes that members of the CTC can negotiate per diem rates of $0.50 or less for barrier facilities. This annual cost is reasonable for a massive resource with such broad utility, but funding will nonetheless require unequivocal and international support, especially from groups studying transgenic, knockout, and mutagenized mice. A strong feature of the cross is that it complements and extends research carried out using KO, TG, and ENU mice by making it possible to systematically explore strong-by-weak allele interactions (see **Point 4**, above).

6. Gene-by-environment interactions. Gene-by-environment interaction (GXE) is a crucial problem in genetics that has been difficult to study in any mammalian population. In the last year gene-by-toxin interactions have also unfortunately become far more important. GXE analysis requires the use of isogenic lines that can be studied in large numbers in different environments and usually over a period of years. Mouse experimental geneticists usually have adequate control over many environmental factors, but have not had sufficiently large isogenic mapping panels. The Collaborative Cross solves this problem. The load of QTLs mapped under one set of environmental conditions can be supplemented by a new set of QTLs mapped under a different set of environmental conditions.

7. Analysis of genetic correlations. The CTC will collect, curate, and distribute large amounts of phenotype data for the collaborative mapping panel. A major enticement to motivate the adoption of the collaborative mapping panel will be to

acquire high quality data on the transcriptome, proteome, and metabolome of 10 to 25 key organ systems in adults of both sexes. The number of RI and RIX lines that can be systematically phenotyped as part of this effort will depend on scientific motivation and funds. High throughput array and proteome methods will be much more practical by the time the RI set is ready for use and distribution starting in 2006–2008. Our intent is to ensure that all investigations of complex traits begin with solid QTL data for large populations of transcripts and proteins in several major organ systems.

8. Freedom from genotyping. The CTC will be responsible for genotyping the collaborative mapping panel. The resource will initially be genotyped at a marker density sufficient to place all breakpoints into 1-Mb bins. All breakpoints will eventually be located as precisely as possible given markers and haplotype diversity. Ideally, we would achieve a resolution of better than 0.25 millimorgans; equivalent to 100 kilobases and a separation of 1–5 genes (*Fig. 3*). Ultimately, each of the parental strains will need to be sequenced either selectively or completely. Sequencing needs to be completed as soon as possible after the stage at which RI strains are available in large numbers.

9. Unrestricted access. Use of strains of mice, tissues, genotypes, and community-acquired phenotypes should be unrestricted for public use. All CTC-funded resources must be available without restriction, but with adequate cost recovery and subject to availability. In the case of resource limits and conflicts, a CTC subcommittee will rank requests and devise ways to share material and mice more effectively. Likewise, the resource should be useable by labs that do not have access to large mouse facilities. This will be accomplished through resource populations maintained at distribution research centers that will also provide visiting research teams access and equipment for phenotyping resource populations. It is crucial that all CTC RI strains be part of a single publicly available resource without legal restriction. The SNP Consortium's solution to the issue of ownership and rights will be a useful model to study.

During the planning of the Collaborative Cross we considered using up to 16 strains. This type of cross could encompass up to twice the number of rare alleles and haplotypes and provide much greater assurance of capturing moderate frequency alleles (due to sampling error some common alleles will be unsampled even in an 8-way cross). The difficulties in producing a 16 way cross are substantial and the increase in cost is exponential. We argue that by carefully selecting the 8

way cross, we can meet our goal of substantial genetic diversity in a cost-effective manner.

The case for including sequenced strains. The genome of strain C57BL/6J has recently been sequenced as part of a public and international effort. Partial sequence data are also available commercially and with considerable restrictions for A/J, DBA/2J, and 129 from Celera Genomics. BAC libraries will shortly be available publicly for A/J, CAST/Ei, and DBA/2J (see Table 1). It is a given that C57BL/6J will be included in the 8-way cross, but the other partially sequenced strains will have to compete for a place in the cross on the basis of genetic diversity. While there are obvious advantages of including strains that have been sequenced, or for which there are currently large numbers of known alleles at SNPs, we believe that for a resource with an expected utility of many decades, foremost consideration needs to be give to haplotype diversity rather than current molecular data and reagents.

The importance of complete strain haplotypes. The genomes of several of the most common inbred strains consist of haplotypes that trace back to the two most prevalent sub-species of *Mus musculus—M. m. musculus* and *M. m. domesticus*. Wade and colleagues (2002) estimate that haplotypes in common inbred strains extend an average of 1.2 megabase. Their genomes should therefore consist of ~2200 alternating *M* and *D* blocks. Eight strains will collectively archive 16,000–18,000 breakpoints, adding significantly to the 100,000 recombinations we will accumulate during the production of the Collaborative Cross. To exploit these historical breakpoints will require extremely high density genotyping of SNPs and microsatellites (50–100 kbp resolution). By characterizing

Table 1. Strains to consider for a multiway RI set (also see Appendix *figure 6*)

Strain	Comment
129S1/SvImJ	standard (Castle strain)
A/J	standard (Castle strain), BAC library by de Jong
AKR/J	standard (Castle strain)
BALB/cByJ	standard (Castle strain)
BTBR	standard (other origin)
C3H/HeJ	standard (Castle strain)
C57BL/6J	standard (C57 strain), full 7x genome sequence
CAST/Ei	*M. m. castaneus* (outlier; wild-derived) BAC library
CBA/J	standard (Castle strain)
CE/J	
CZECHII/Ei	wild *M.*
DBA/2J	standard (Castle strain), BAC library by Rod Wing
FVB/NJ	standard (Swiss strain)
I/LnJ	standard (Castle strain)
JFI/Ms	*M. m. molossinus* subspecies (wild-derived strain)
KK/H1J	standard (China/Japan strain)
LG/J	standard (Swiss strain?)
LP/J	standard (Castle strain)
MA	
M16i	D. Pomp selection line (Swiss strain)
MOLF/Ei	*M. m. molossinus* subspecies (wild-derived strain)
MSM?Ms	
NOD/LtJ	standard (immune defects0
NZB/BINJ	standard
NZO/W	standard
PANCEVO/Ei	wild *M. spicilegus* (outlier test; Wild-derived strain)
PERA/CamRk	wild *M. m. domesticus* (wild-derived strain)
PERC/Ei	wild *M. m. domesticus* (wild-derived strain)
PL/J	standard (other origin)
PWD	wild *M. m. musculus* (wild-derived strain)
PWK/Ph	wild *M. m. musculus* (wild-derived strain)
RIIIS/J	standard
SKIVE/Ei	wild *M.*
SJL/J	standard (Swiss strain)
SM/J	wild *M. m. domesticus* (Castle strain)
SPRET/Ei	wild *M. spretus* (different species; extreme outlier)
SWR/J	standard (Swiss strain)
TIRANO/Ei	wild *M*
WSB/Ei	wild *M. m. domesticus* (wild-derived strain)
ZALENDE/Ei	wild *M*

Notes of Strain Table. We have included a table of strains that we are now genotyping at 500 new sequence-derived microsatellites. For strain data see *www.informatics.jax.org/external/festing/search_form.cgi* and *http://jaxmice.jax.org/jaxmicedb/html/wild.shtml*

polymorphisms in a large set of inbred strains we can use rigorous quantitative criteria to select eight strains that incorporate a diverse and balanced set of ancestral haplotypes (Table 1). By sampling a wide variety of mice, we expect to be able to recover inbred strains that collectively harbor four major ancestral haplotypes; doubling the genetic diversity now harbored in the most common inbred strains.

PART II. IMPLEMENTING THE CROSS

We hope to choose strains for the RI cross before the end of 2002. These strains are labeled A through H in this summary. We use specific crosses in these examples (A x B; EF x GH), but a large number of unique permutations of parental strains will be generated. The generation numbers of adults in each step are labeled as **G** numbers.

Synopsis. The simple but powerful design of this cross, outlined below and diagramed in *figure 2*, ensures that all RI strains are independent in the sense that they do not share recombinations that are identical by descent. RI strains can be generated efficiently at several breeding centers without transport of stock. Starting material for each RI strain consists of four F1 intercrosses. These four F1s are intercrossed in succession producing a breeding funnel in which the parental haplotypes are merged and recombined. Each funnel is an independent and easily replicated process. Our tactic has been to minimize cost and cage counts throughout while generating a 1K RI set. We will not struggle to maintain strains with low production. In order to compensate for inevitable attrition, we plan to initiate ~35% more strains than we anticipate being able to retain.

How many strains? We are committed to retaining a final set of at least 256 RI strains available promptly from live stock. While this number is sufficient for mapping additive effect QTLs with unprecedented precision, it is not sufficient for the systematic dissection of 2- or 3-way epistatic interactions—a long term goal of many CTC members. For studies at this level, a 1K set of RI strains will be required. From a logistical perspective there are no special organization problems in generating, genotyping, or maintained a 1K RI set, although ensuring that the set is secure (duplicate colonies or adequate cryopreserved stock) is vital. The costs of maintaining sets at two sites would be approximately $1.6 million/year.

G0. Parental Strains. We will cryopreserve embryos of the eight parental strains within the first year of project initiation. We must preserve very recent F-number stock. This is neces-

sary because strains will drift slowly away from year 2003 founder stock due to the accumulation and fixation of novel mutations. Without frozen founder stock we will have difficulty adding new strains without introducing systematic subset differences. For example, the original 26 BXD RI strains introduced in the late 1970s differ as a group from the new set of 10 BXD strains introduced in 1999 for reasons that probably involve the fixation of new alleles in C57BL/6J and DBA/2J over a 20-year interval.

G1. Production of F1. We will produce a full diallel cross of the eight strains. Imagine an 8 x 8 chess board in which each of the squares is populated by an F1 intercross and the diagonal consists of parental strains. A total of 56 F1s (28 reciprocal pairs) will be made in one or two locations. The F1 animals will be shipped as breeding trio (1 AXB male with 2 CxD females) and the pair of triplets required to produce an 8-way RI line will be shipped simultaneously to major breeding centers (see Fig. 2, ABxCD box shipped as one trio). The costs for this step are reasonable: assuming six of each reciprocal breeding pairs per cross, the animal costs are ~$5,000. Cage costs of maintaining the parents and the offspring (until 7-weeks-of-age) are ~$25,000. Shipping costs are ~$20,000. Approximately 5,000 F1 offspring will be required. This first step could begin as early as July 2003. The CTC will coordinate distribution of F1s.

> **Use of F1 animals.** The isogenic F1 progeny are a valuable resource in their own right and they can be used for in-depth phenotyping and mapping efforts in 2003 and 2004. These animals do not incorporate any breakpoints and have an inbreeding coefficient of 0. However, with a dense haplotype map of the eight parental strains, the diallel progeny may be used for some types of mapping, although their primary function will be to study genetic architecture more broadly as well as maternal and cytoplasmic effects. The diallel is not intensive in terms of cage requirements. We will need a minimum of 30 males and 30 females from each F1 to populate the G2 cages in the next step of this cross. To generate 30 animals of each sex roughly at the same time will require setting up 10 to 20 cages per cross: approximately 750 cages total.

The simple starting structure of the cross makes it feasible for several international sites to contribute to the initiation and progression of the cross. CTC coordination will involve subdivision of breeding effort at steps G2, G3, etc. Colony informatics, phenotyping, and genotyping will need to be tightly coordinated. Friendly competition and collaborative help should improve morale and performance. This will also provide insurance against colony infection or other catastrophes.

In general, our workgroup supports and encourages efforts to ensure international participation in the CTC resource development. One caveat is that all sites (US and elsewhere) should have the necessary cage capacity to place stock behind a pathogen barrier once the inbreeding begins (see below). We discussed distributing efforts across 4–6 major sites. This number of centers would provide adequate dispersion of animals, expertise, and interest, and would also protect the effort.

G2. Production of a G2 (a 2-way by 2-way cross). The 56 types of F1 progeny produced in the first generation (G1) will be intercrossed. AB will be crossed to CD and to DC; BA will be crossed to DC and CD, etc. There are a maximum of 3080 possible pairings of F1s, but we will only generate the subset of non-intersecting pairs that have no shared parental strains (we will not cross between AB with either BC or CB, because the B haplotype is shared). There are a maximum of 56 x 30 or 1680 of these non-intersecting but reciprocal pairings. A color matrix illustration of the 1680 possible ABxCD crosses is appended at the end of this report (Fig. 4). This complete coverage ensures a balanced representation of the sex chromosomes and of all haplotypes in all combinations. Single chromosomes of G2 progeny will have AxB recombinations or CxD recombinations (not both) on a subset of chromosomes.

At this point the major breeding sites must be chosen and committed to six years of developing the RI strains. For example, four sites might each be responsible for 420 strains. Each site would need approximately 3x this number of cages for breeding. We are assuming a cost of no more than $0.60 per diem. Cage costs at four sites would be approximately <$300K/year. These costs should stay relatively constant over the period of RI development as increasing per diem costs will be offset by decreases in the population size. Even with losses of 8% per year, a final sample of 1K RI strains will be obtained.

Selection of G2. (This is an optional step indicated to the left side of *Fig. 2*.) Roughly 20–30% of chromosomes from a random sample of G2 individuals will be non-recombinant. Animals will inherit numerous chromosomes that have *A*-only, *B*-only, *C*-only, or *D*-only haplotypes; see recent papers by Broman and colleagues (2002, complextrait.org/ctcsche/mousebcpapers.pdf), Williams and colleagues (2001). To select against these less informative non-recombinant chromosomes and to increase the recombination density in the final RI strains, we may genotype G2 animals at two to four markers per chromosome (up to 60 genotypes per genome) and select the more highly recombinant individuals. We will select from among progeny of two to four litters (18 to 45 mice). It will usually be possible to select G2 animals that harbor approximately 30–32 recombinations: 15–16 per parental gamete. Because

animals will be genotyped as part of this selection process, these G2 animals would be an extremely valuable mapping resource.

All of the 4-way G2 animals that are genotyped can used for phenotyping by CTC investigators and other collaborators. These animals will have chromosomes that are AxB and CxD recombinants, and will be different from a conventional F2 in several important respects: 1. There are four genotypes at any one marker (A-C, A-D, B-C, B-D), all at close to a p of 0.25. 2. Animals have a formal inbreeding coefficient of 0. However, the ancestral haplotypes of strains A, B, C, and D will often be common, and there will therefore be numerous regions that are identical (homozygous) by ancient descent.

Breeding note. The total load of recombinations in each fully inbred RI strains is influenced by the selection coefficient for recombination events in this and the next two generations. Selecting the most recombinant 10% of animals can increase the final load of recombinations more than 1.4X over random expectation. However, the benefits of selection need to be considered in light of a cost of genotyping (approximately $400,000 per generation, see below). Funds can either be expended to optimize recombinations in the final generation or to generate and house larger numbers of strains. Generating a single RI strain has a total cost of approximately $5,000, and 80 strains could be generated for the cost of a single generation of genotyping.

Genotyping note. To select the most recombinant of the G2 progeny would require a relatively intense genotyping effort. Simulations have shown that for genotyping and selection to be moderately effective, we would need to generate ~20 animals per AB x CD cross and select the single most recombinant male and female (K. Broman, unpublished). This 10% selection coefficient would require at a minimum genotyping of 1680 crosses x 20 animals x 20 chromosomes x 2 markers (approximately 1.5 million genotypes). This work will need to be accomplished over a 150-day period, or roughly 10,000 genotypes per day. The scientific merits of this work must be balanced against the cost of $400,000 for genotyping. We assume a total cost of approximately $0.25/genotype for a project of this size (c.f. James Weber; current genotyping costs of the Marshfield Mammalian Genotyping Center).

Design note. We considered the possibility of intercrossing ABF1 animals to produce F2, F3, or even F4 populations. F4 advanced intercross animals would incorporate 2x the recombination load of a conventional ABF2 and they could be generated without any shared breakpoints. In principle, such F3 or F4 animals could be used to initiate the 1680 G2 strains above. For several reasons, we have decided against this method in favor of an immediate cross of ABF1 x CDF1 with subsequent selection for the most highly recombinant individuals. This simple alternative avoids matings that produce partly inbred individuals and with sufficiently intense selection can generate comparable recombination densities while ensuring balanced representation of parental alleles in a shorter period of time.

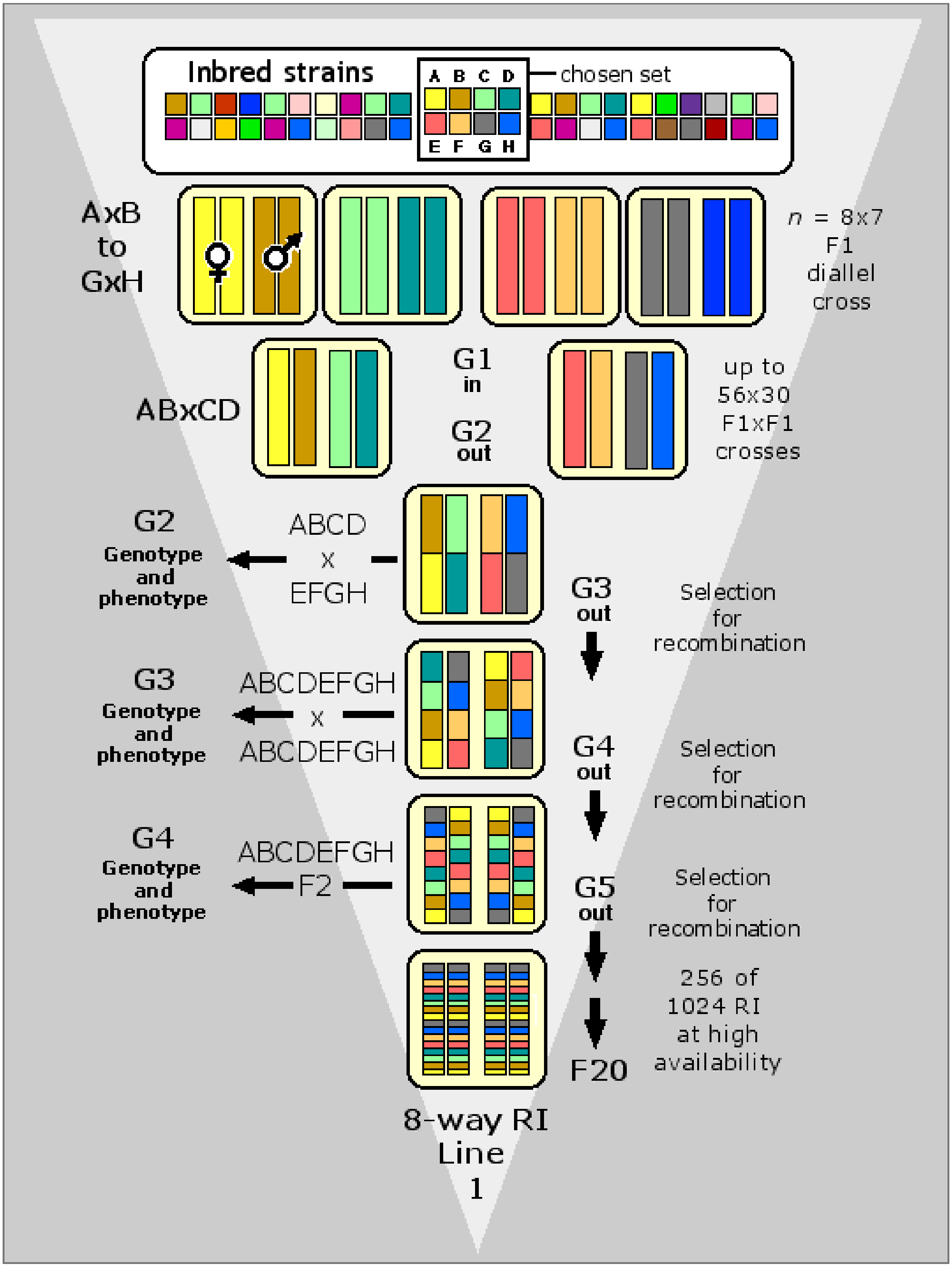

Figure 2. Outline of the production of a single 8-way RI strain. See text for details.

G3. Production of G3 (4-way by 4-way). ABCD will be crossed to EFGH to generate G3 offspring. The number of ways to breed non-intersecting 4-way G2 parents is 8-factorial (40,320), but we will limit the G3 to a balanced sub set of 1680 strains (e.g., ABCD to EFGH; ABEF to CDGH). As with the G2 production, cage space will be limited at the breeding sites and it is planned to only fund the production of a single G3 litter. Again we will encourage CTC investigators and other potential collaborators to make use of this unusual G3 resource that should be available beginning in July of 2004.

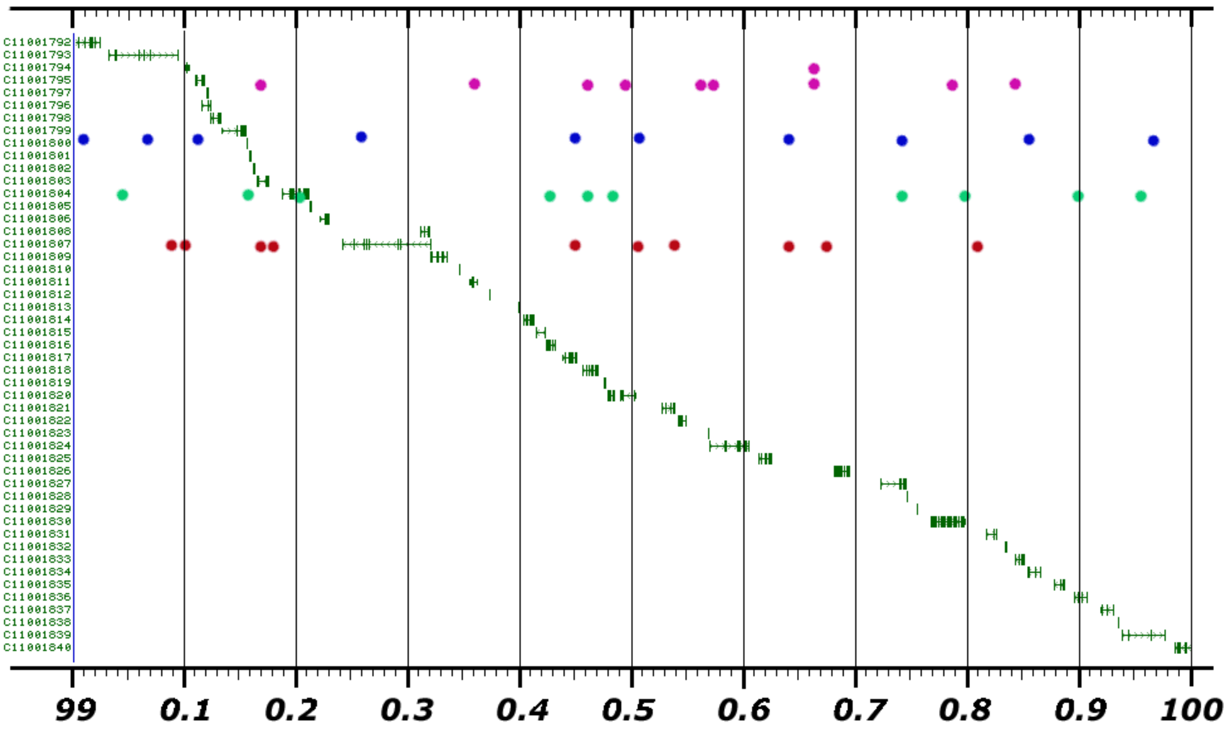

Figure 3. Modeling the distribution of recombination events across a 1 Mb interval of Chr 11 that contains approximately 49 genes (*diagonally offset lines* listed in the *left column*). Gene density in this region is roughly three-fold higher than average for the whole genome (see *http://genome.ucsc.edu*). Each *row of spots* near the top gives the location of 10 randomly generated recombination events along this 1 Mb (0.5 cM) interval. A set of 256 8-way RI strains will typically harbor ~26000 recombinations across a 2600 Mb genome—a sufficient number to generate one row's worth of recombinations. A set of 1024 RI strains will incorporate all four rows of recombinations. While the number of genes between recombinations averages ~5 per row, the Poisson distribution of these events leads to intervals as large as 200 Kb. Even with the full set of 40 recombinations, only 20–25% of genes are isolated unambiguously between flanking recombinations. With 40 recombinations per 1 MB there are still intervals of 100 Kb between recombinations. The model assumes random recombination (no hotspots) and perfectly informative markers that can resolve recombination events with a precision of ~10 kb. Both assumptions lead to a best-case assessment of mapping precision. This simulation emphasize that a 1K RI set is not overpowered, particularly for mapping QTLs that to fall in gene-rich regions. The simulation does not factor in the historical haplotype breakpoints that will add roughly another row of breakpoints.

Optional Genotyping*.* Ends and middles of chromosomes could again be genotyped to recover those G3 animals with higher numbers of crossovers. Phenotypes of these animals and of the previous G2 would be pooled. The total G2–G3 mapping resource would contain a very large number of animals (about 20 times the number of RI lineages that we propose to make, or as many as 1680 x 20). Single chromosomes of the G3 animals will often be either (AxB)x(CxD) or (ExF)x(GxH). Single chromosomes will contain haplotypes from one strain (hopefully rare), 2 strains (uncommon), or 4 strains (most common). Selected G3 animals will harbor a total of about 72 recombinations (36 per haplome). Formal inbreeding coefficient is still 0.

Note on gene-by-environment interactions. There will be interesting and systematic differences in phenotypes and QTLs detected in G2 and G3 animals produced at different centers. This should be viewed as a positive feature of the cross design and as an opportunity to systematically explore gene-by-environment interactions. Phenotyping protocols will be coordinated closely, and it will be useful to send some strains and mice between sites for phenotyping and cross-validation analysis.

Optional Genotyping. This generation of animals could also be genotyped at two or three markers per chromosome, and the most recombinant siblings could again be bred. The total estimated number of recombinations incorporated into the genome of each of the G3F1 animals given selection in each of the preceding three generations for the most recombinant 10%–20% of animals is 90–110. (Start date, Oct 2003)

Recombination density of RI and RIX strains. We have estimated that without any selection the final RI strains would typically incorporate between 90 to 100 recombinations per line (94 ± 11 SD; estimate of Churchill, Bulutoglu, Williams). With selection over several generations, we anticipate that this number can be effectively increased to 115 recombinations per RI line or 230 recombinations per RIX line. In comparison the genome of a typical F2 animal contains 30 recombinations, that of an N2 backcross contains 15 recombinations, and that of a standard RI strain contains 54 recombinations.

G4. Production of G3F1 (8-way by 8-way cross). G3 8-way animals produced in the previous generation will be intercrossed. These can be sib matings without any loss of productive recombinations. The G3F1 progeny of sib-mated G3 parents will often have single chromosomes with [(AxB)x(CxD)] x [(ExF)x(GxH)] recombination blocks. These animals will have an inbreeding coefficient of 0.5 and are in many ways like an F2.

Breeding note. Fecundity of these intercross generations will be high. A single cage will often be adequate. However, two to three cages would be helpful to select highly recombinant individual quickly.

G5. Production of G3F2. Second sib mating of 8-way parents.

G6–G13. G3F3 through G3F10. This is the initial critical phase of inbreeding. At F10 the strains should be about 90 to 94% inbred. (through Feb 2006).

Genotyping note*.* At F10 it might be worthwhile to genotype the pools of DNA from 4 to 10 animals at 500 to 1000 markers to gauge the ultimate balance and performance of the whole RI set. These animals would then be used in molecular and metabolomic phenotyping and gene network QTL studies in 2006.

G14–G20. The final stages of inbreeding. We might consider the application of speed inbreeding at this stage with selection against heterozygotes and fixation of alleles and haplotypes that will improve balance of whole set. The efficacy of speed inbreeding will depend primarily on genotyping cost. The gain in recombinations load and genotype balance could be substantial. With speed inbreeding the F15 may be considered fully inbred. (Through the end of 2008).

Strain attrition during inbreeding. We anticipate loss of approximately 30–40% of strains during steps G8 to G13 (F5 through F10 generations of inbreeding). This is consistent with the loss of strains during the production of the 79 strains of LXS RI strains from 125 original strains. If we begin with 1680 G3F2 founders, we should be able to retain approximately 1024 strains. All of these strains, or a carefully selected subset (see below), will be retained as live stock or as cryopreserved stock.

Use of transgenic parental strains to facilitate genetic manipulation of RI lines. We anticipate establishing embryonic stem cell lines from all or a subset of the final RI strains. Given recent successes with establishment of ES cells from a variety of strains, it is likely that a substantial subset of the RIs will produce these cell lines.

Given ES cells from a large number of strains, certain modifications to some or all of the eight founder strains might prove advantageous. Two modifications to be considered are:

1. A floxed reporter gene
2. A *lox* or *frt* site that can serve as a target for insertion of transgenes. This would allow the identical *lox* or *frt*-containing transgene construct (e.g., a BAC) to be inserted at the identical site in multiple different lines to assess effects of genetic background on expression.

An appropriate target site and *frt* construct are available. Given the projected timetable for this project, an existing ESC line derived from C57BL/6J (available from R. Reeves and

colleagues at Johns Hopkins University) could be targeted and appropriate mice made as founders in time to begin the breeding. If two strains were used, 1/8 of the resulting RI lines would be expected to contain the homozygous target site. For C57BL/6J, the strain could be expanded from chimeras using an existing floxed ROSA26 on a C57BL/6J background, thus putting two modifications into the end RI sets.

ES cells from a number of other strains have been reported; their availability and utility are unknown at this point. It might not be practical to reestablish modified strains from these lines.

Use of RI cell line stock. Metabolic assays of cell lines from all RI and many RIX lines will be undertaken using cellular microarrays. Fibroblast cell lines for the complete RI set and up to 10,000 RIX lines will be acquired and stored for high throughput cellular screens. These cultures will be invaluable for studying GXE at the cellular level.

Scientific Use and Analysis

The design of this cross enables CTC members to engage in cutting-edge QTL mapping throughout the project. However, the amount of science done during the production phase is entirely dependent on a significant budget for genotyping one or more intermediate generations. This is a topic that we will want to revisit soon.

Year 01. In year 01 we focus on the phenotypes associated with the 8 inbred strains and the associated 56 reciprocal F1 intercrosses. This is essentially a phenome project of vigorous non-inbred but isogenic strains of mice for which we expect to have superb haplotype maps. The intent here is not to duplicate the efforts of the mouse inbred strain phenome project but rather to supplement this effort, especially for phenotypes not currently included in the database. One such area would be the development of a transcriptome database for various tissues. Once the inbred strain database is developed, attention should turn to the F1 intercrosses, which can be easily recreated at numerous sites. Issues of dominance, maternal effects and so on are easily measured. The quantitative genetics core of the consortium will be responsible for helping investigators analyze these data and for developing new analytical tools. These services will be made available to all investigators studying the eight strains and associated crosses used to form the RI panel.

Year 02-05. Collaborative QTL mapping using G2 through G6 individuals. The data obtained from the analysis of the eight inbred strains and the reciprocal F1 crosses will generate numerous hypotheses as to phenotypic distribution among

the 4-way crosses. As noted previously, the number of 4-way animals that would be available to a single investigator may be limited; however, sufficient numbers should be available to obtain strong preliminary data to justify recreating crosses of interest. A similar argument applies to moving from the 4-way to the 8-way crosses. This point is important since it will provide a foundation for understanding the genetic architecture of the final RI strains. This effort will segue perfectly with the eventual analysis of RI and RIX strains.

Year 05 to 07. Analysis of near isogenic RI strains with inbreeding coefficients above 0.95. The near isogenic strains will be particularly interesting, especially in view of the fact that the precise locations of many QTLs will already be known. Any QTL located in a region that is still segregating can be converted into sibling strain pairs in which the QTL is fixed for alternate alleles. This method can be used to maximize recombinations and to explore QTL effects.

Year 07 onward. Start of systematic phenotyping of fully inbred RI and the associated RIX crosses. Of the expected 1K strains that will be viable at year 07, those strains showing the highest level of recombination will be chosen for the core set of 256.

PART III. HOUSING, DISTRIBUTION, and ARCHIVING

Which Strains to Keep. A core set of 256 RI strains will be chosen as the consensus mapping resource set and maintained at high availability. This set and derivative RIX strains will be optimized for recombination load and distribution in gene-rich regions and for comparatively balanced representations of the eight haplotypes and alleles. The core set of RI strains will be housed and bred at several international sites. The full 1K set will be retained at maintenance level (~6 cages/line). The 1K set will be used for systematic high-throughput molecular phenotyping at major centers and for final mapping projects that require the ultimate resolution. Strains with reproductive problems will be cryopreserved. Prior to cryopreservation, we will collect tissue for an archive of normal adult RI and RIX tissues.

Distribution of RIX strains. We would like to encourage scientists to exploit the non-inbred RIX derivatives of the RI strains. RIX strains provide users with twice the haplotype diversity and twice the load of recombinations of an equal number of RI strains, with lower within-line variances, with access to dominance signal, with reduced sensitivity to collateral damage from recessive alleles (e.g., blindness in

behavioral studies), and with the ability to assess parental effects. Viability and robustness of RIX progeny is also better, and we will be able to provide and distribute them more inexpensively to researchers. The RIX progeny of an 8-way cross have an inbreeding coefficient that is essentially zero. They are good mouse mimics of admixed human populations. We recognize that for some applications, even 256 RI strains will be statistically limiting. In some situations, the RIX crosses can overcome this limitation.

Organization of the Consortium (still preliminary)

Production. As noted previously, the production of the RI strains will be confined to four to six large centers each of which will be capable of devoting a minimum of 2000 cages to this project for a period of no less than five years. All databases will be maintained centrally, with local mirrors. Once the first phase is complete (the RI strains are formed), demand is expected to justify the need for four distribution centers, at least one of which should be overseas. A single center will be needed to handle the cryopreservation of the strains – this need not be one of the production centers.

Informatics Core. There are several significant informatics components. These components include coordination of breeding, data repositories, the development of data mining tools and outreach to the larger genetics community. All efforts will be implemented (when possible) using open source software (e.g., PostgreSQL, MySQL, PHP, Perl, Python, R and R/qtl, XML). For this reason, we encourage development using a portable programming style. The key features of the informatics core include:

A. Breeding schema testing and colony operations database operations (now in progress).

B. High-throughput fast-turnaround genotyping cores and informatics resources to ensure optimal breeding.

C. Final genotyping informatics of RI output.

D. High-level analysis of genome-type structure of the entire RI set to select optimal set of strains as a consensus panel using ‘minimum aberration’ and ‘maximum unconfounding’ design criteria to optimize simultaneously for both additive effects and 2-way interactions (www.cs.auckland.ac.nz/~philip/Stats/stexdes.html#2a).

E. Phenotype database development and web interface. Transcriptome-QTL database development. Proteomics and metabolomic informatics.

F. Order, tracking, and distribution database system.

G. Automatic and guided on-line QTL analysis using hybrids of WebQTL and R/qtl. Focus of this system is on end-user tools and interface. The system will need to deal with this particular complex cross, multiple-trait mapping, automatic permutation analysis, and will have to be able to handle and display a wide variety of phenotypes and their QTLs. One important product of the CTC should be the definition of precisely mapping QTGs that are ready to take into the laboratory for experimental studies.

Quantitative Genetics Analysis Core. This core will have the responsibility for statistical genomic tool development. This will include automatic and guided on-line QTL analysis using hybrids of WebQTL and R/qtl with a focus on end-user tools and interface. Areas of emphasis will include complex cross analysis, multiple-trait mapping, automatic permutation analysis, bootstrapping to estimate support intervals, and sophisticated procedures to detect epistatic interactions. Software tools will need to handle and display a wide variety of phenotypes and their QTLs. This core should also focus on developing new computational tools to reach deeper into multidimensional data sets (sequence, genotype, haplotypes, phenotypes, and environments/pathogens).

Administrative Core. The administrative core will be responsible for the day-to-day operation of the consortium and will be responsible for coordinating regular meetings of participating groups, including internal and external scientific advisory panels.

Genotyping/Sequencing Core(s). Although these cores could be separate, it seems likely that their integration will facilitate the development of the required databases. The initial goal of the Genotyping Core is to locate recombination breakpoints in all RI strains with a precision of approximately 1 Mb. This will require 10,000 or more markers, many of which will have to be custom generated to distinguish alleles and haplotypes over short intervals. With full genome sequence data, it is now possible to rapidly and selectively expand the current set of ~6200 MIT dinucleotide markers. SNP databases are now expanding rapidly and we expect to have more than adequate resources to define breakpoints into 1 Mb bins in the final stages of inbreeding. Since each line will incorporate ~100 breakpoints—typically 3 to 10 per chromosome—it should be possible to achieve high resolution maps using only a small fraction of the available markers. It is likely that SNP genotyping protocols will be sufficiently advanced and inexpensive to achieve 100 kb resolution for the majority of breakpoints.

The goal of the sequencing core is to sequence parental strains to define the majority of polymorphisms that potentially contribute to trait variance. Resequencing could be highly selective, with a focus on promoters and exons. A 0.1X coverage might be adequate.

Phenotyping Cores. As the RI strains are developed, it is proposed that a number of specialized phenotyping cores should come online to facilitate the analysis of the large RI panel. The ability to analyze 256 RI strains and perhaps an equal number of RIX crosses, even if only a few animals/group are needed, will require resources (in terms of space and equipment) on a much larger scale then is usually needed. These should include a metabolic core, a behavioral core, a cancer core and a histology core. In addition, there will be a need for at least one core with sufficient space to allow visiting investigators engage in collaborative phenotyping. These cores will not be part of the founding application but it is assumed that the funding institutions will be committed to their development.

References

Belknap J (1998) Effect of within-strain sample size on QTL detection and mapping using recombinant inbred mouse strains. Behav Genet 1998 28:29-38.

Broman KW, Rowe LB, Churchill GA, Paigen K (2002) Crossover interference in the mouse. Genetics 160:1123–1131. Online at www.complextrait.org

Darvasi A (1998) Experimental strategies for the genetic dissection of complex traits in animal models. Nat Gen 18:19–24.

Gibson G, Mackay TFC (2002) Enabling population and quantitative genomics. Genet Res Camb 80:1–6.

Hitzemann RW, Malmanger B, Cooper S, Coulombe S, Reed C, Demarest K, Koyner J, Cipp L, Flint J, Talbot C, Rademacher B, Buck K, McCaughran Jr. J (2002) Multiple cross mapping (MCM) markedly improves the localization of a QTL for ethanol-induced activation. Genes, Brain and Behav 1: 214–222.

Hunter KW, Williams RW (2002) Complexities of cancer research: mouse genetic models. ILAR J 43:80–88. Online at www.complextrait.org

Felsenstein J (1989) PHYLIP—Phylogeny inference package (v. 3.2). Cladistics 5:164–166.

Schalkwyk LC, Jung M, Daser M, Weiher M, Walter J, Himmelbauer H and Lehrach H (1999) Panel of microsatellite markers for whole-genome scans and radiation hybrid mapping and a mouse family tree. Genome Res 9:878-887.

Strachan T, Reed AP (1996) Human molecular genetic. Wiley-Liss, New York.

Threadgill DW, Hunter KW, Williams RW (2002) Genetic dissection of complex and quantitative traits: from fantasy to reality via a community effort, Mamm Gen 13:175–178. Online at www.complextrait.org

Williams RW, Gu J, Qi S, Lu L (2001) The genetic structure of recombinant inbred mice: high-resolution consensus maps for complex trait analysis. Genome Biol. 2: RESEARCH0046.

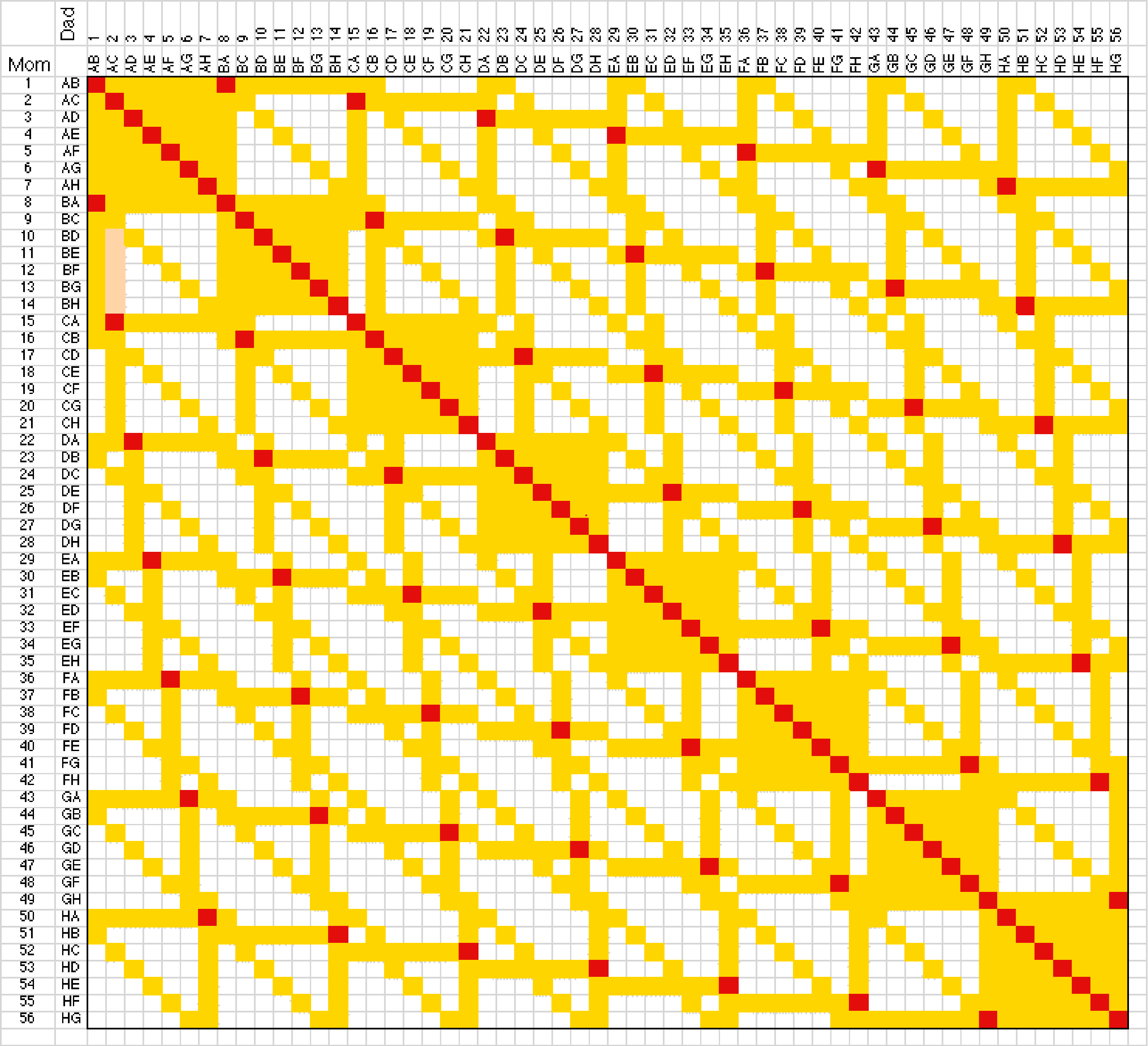

Appendix Figure 4. Illustration of ABxCD-type crosses that can be used to generate as many as 1680 non-intersecting G2 strains. The white 'pixels' represent those crosses that would be generated collectively at a set of CTC breeding centers. Note that across each row, sets of mice are divisible in units of five strains sharing one haplotype.

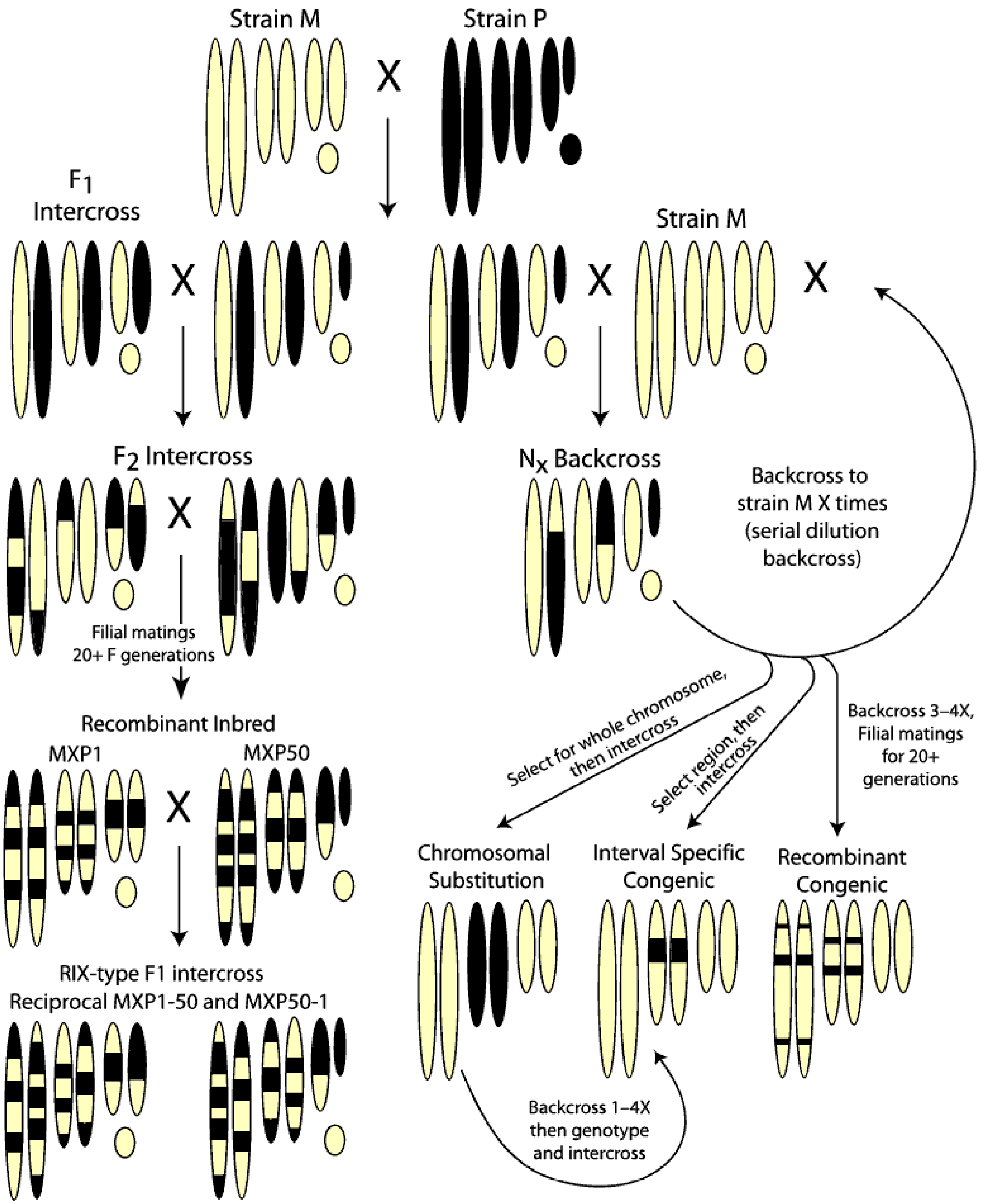

Appendix Figure 5. Experimental crosses. Two pairs of autosomes, chromosomes X and Y, and the mitochondrial genome (small oval) are illustrated. The *left side* illustrates the path leading to RI strains and RIX lines. The *right side* illustrates breeding techniques that simplify the mouse genome by retaining subsets of recombinations and introgressing chromosomal segments from a donor to a recipient strain (paternal line *P* = black; maternal line *M* = light). Hunter and Williams (2001).

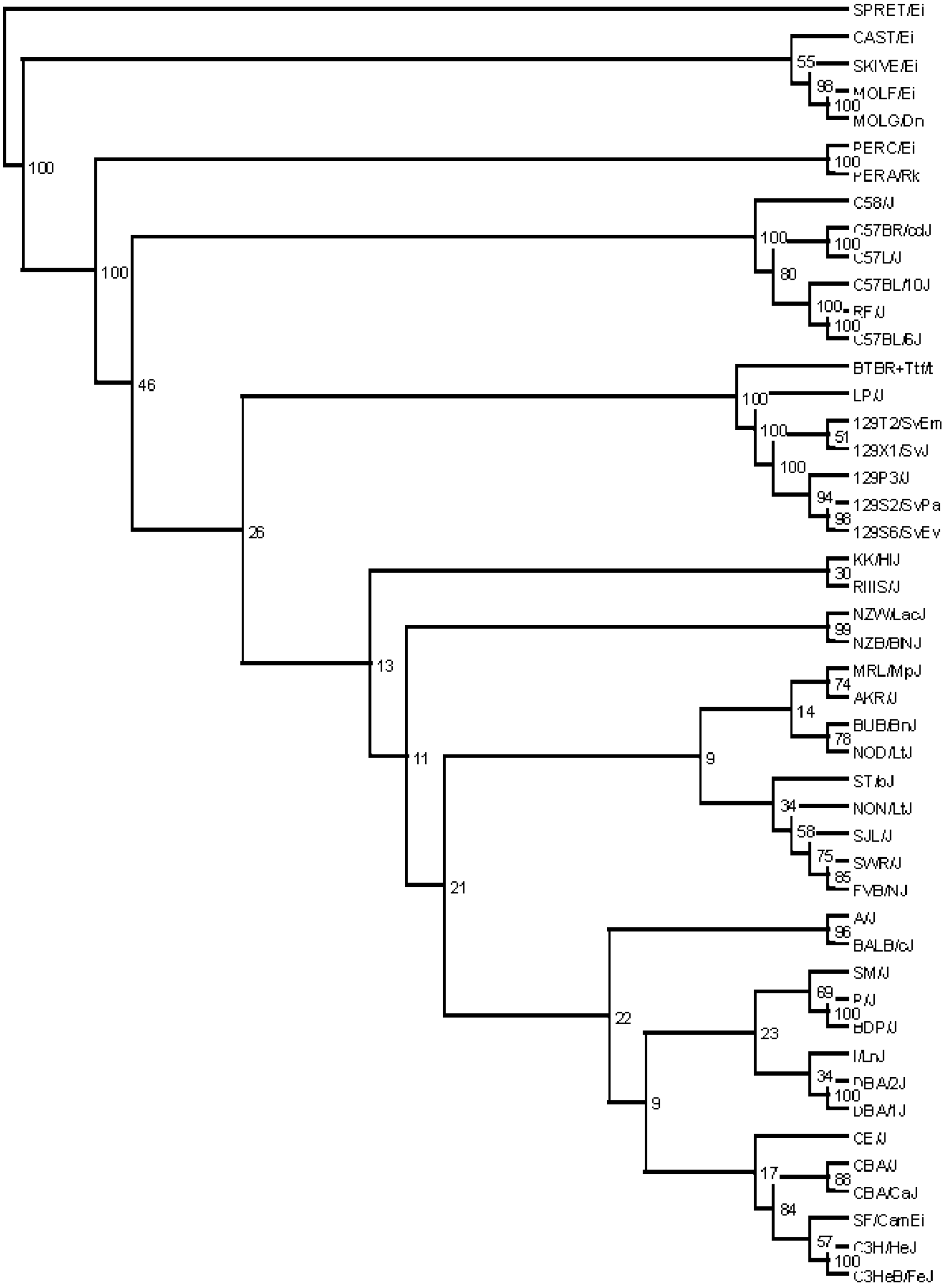

Appendix Figure 6. Wagner parsimony analysis using MIX (Felsenstein 1989) of microsatellite data (298 loci from all 19 autosomes and Chr X) on 48 strains. Genotypes were transformed into binary characters using the technique of Schalkwyk et al. (1999) with SPRET/Ei as an outgroup. Internal figures are the number of bootstrap replicates out of 100 supporting each group. The overall topology agrees with Schalkwyk (1999) except that the C57 and 129 groups are reversed.

Author Affiliations and contact information

Robert W. Williams Ph.D. (corresponding author)
Center of Genomics and Bioinformatics
University of Tennessee Health Science Center
855 Monroe Avenue
Memphis TN 38163 USA
Phone 901 488-7018
Email rwilliam@nb.utmem.edu

Karl W. Broman Ph.D.
Department of Biostatistics
Johns Hopkins University
Baltimore, MD 21205-2105 USA
Phone 410 614-9408
kbroman@athena.biostat.jhsph.edu
http://biosun01.biostat.jhsph.edu/~kbroman

James M. Cheverud Ph.D.
Department of Anatomy and Neurobiology
Washington University School of Medicine
660 S. Euclid Ave.
St. Louis, MO 63110 USA
Phone: 314-362-4188
Email cheverud@pcg.wustl.edu.

Gary A. Churchill Ph.D.
Jackson Laboratory
600 Main Street
Bar Harbor, ME 04609 USA
Phone 207 288-6189
Email garyc@jax.org
http://www.jax.org/research/churchill

Robert W. Hitzemann Ph.D.
Department of Behavioral Neuroscience
Oregon Health Sciences University
3181 S.W. Sam Jackson Park Road
Portland, OR 97201-3098 USA
Phone 503 402-2858
Email hitzeman@ohsu.edu

Kent W. Hunter Ph.D.
Laboratory of Population Genetics
DCEG/NCI/NIH
41 Library Drive
Bethesda, MD 20892 USA
Phone 301 435-8957
Email hunterk@mail.nih.gov

John D. Mountz Ph.D. M.D.
Professor of Medicine
Department of Medicine
473 LHRB, 701 S. 19th St

Birmingham AL, 35294 USA
Phone 205-934-8909
Email john.mountz@ccc.uab.edu

Daniel Pomp Ph.D.
Department of Animal Science
University of Nebraska-Lincoln
Lincoln, NE 68583-0908 USA
Phone 402 472-6416
EMAIL dpomp@unl.edu
http://www.ianr.unl.edu/ianr/anisci/pomplab/

Roger H. Reeves Ph.D.
Department of Physiology
Johns Hopkins University
725 North Wolfe Street
Baltimore, MD 21205-2105 USA
Phone 410 955-6621
Email rreeves@welch.jhu.edu
http://inertia.bs.jhmi.edu/roger/

Leonard C Schalkwyk Ph.D.
Social, Genetic, and Developmental Psychiatry
Research Centre
PO82, Institute of Psychiatry, King's College London
De Crespigny Park
London SE5 8AF United Kingdom
Phone +44 (0) 20 7848 0279
Email l.schalkwyk@iop.kcl.ac.uk
http://www.iop.kcl.ac.uk/iop/Departments
/SGDPsy/staff/senior/LS/home.stm

David W. Threadgill Ph.D.
Department of Genetics, Campus Box 7264
Lineberger Cancer Center
University of North Carolina
Chapel Hill, NC 27599-7264 USA
Phone 919 843-6472
Email dwt@med.unc.edu
http://152.19.39.101

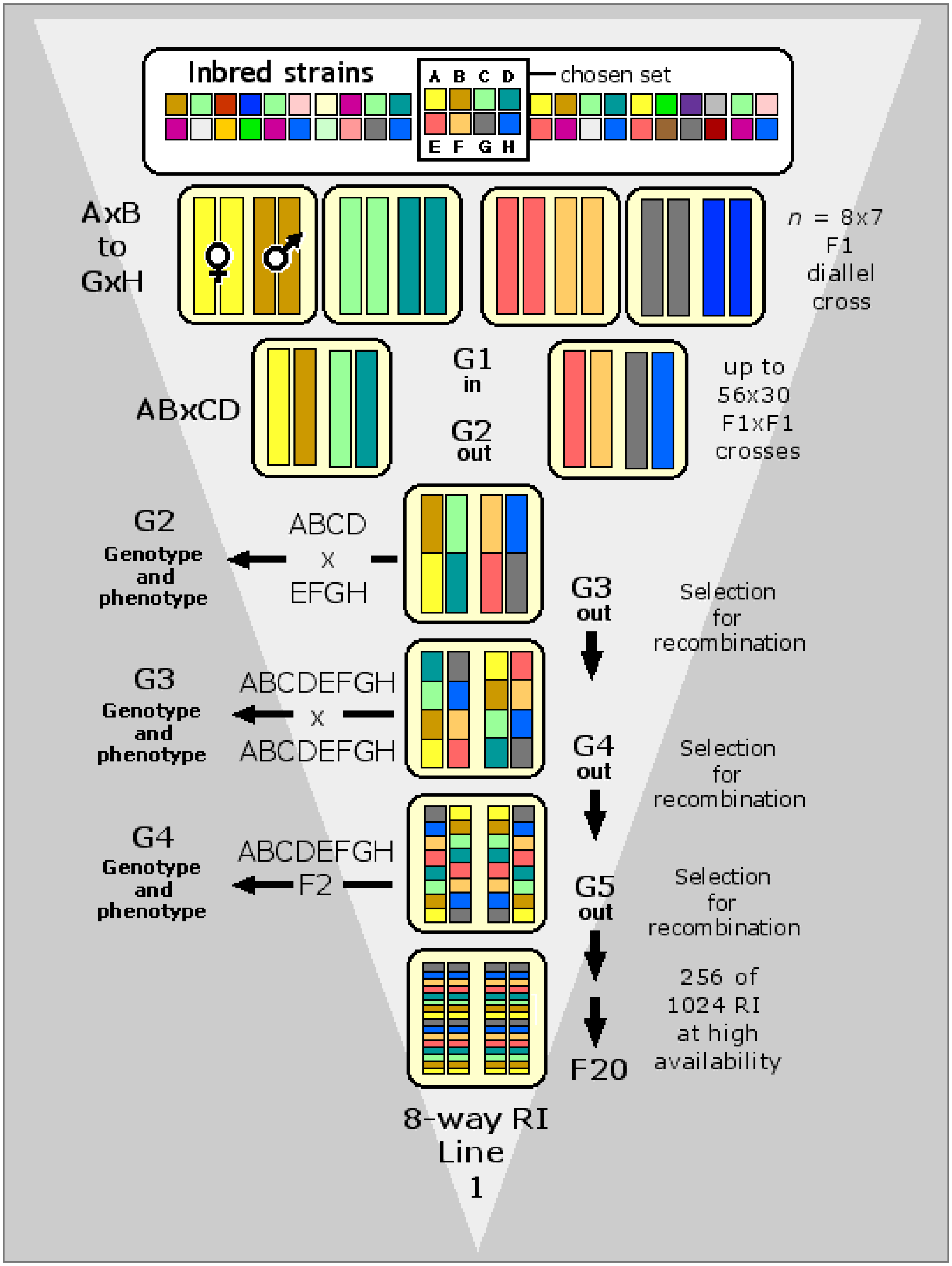

Supported by The Human Brain Project and the Informatics Center for Mouse Neurogenetics, a Human Brain Project/Neuroinformatics program funded jointly by the National Institute of Mental Health, National Institute on Drug Abuse, and the National Science Foundation (P20-MH 62009). *www.mbl.org*
